# Supplementary material for: Chromothripsis during telomere crisis is independent of NHEJ, and consistent with a replicative origin
Source: Genome Res. 2019 May;29(5):737–49. doi: 10.1101/gr.240705.118 (PMC6499312; doi:10.1101/gr.240705.118)
Supplement: Supplemental Material [file supp_gr.240705.118_Supplemental_file_1.zip › contigs/annotated_contigs/DB51/contig.3.DB51_length_749_mean_cov_10.6608811749.docx]

**DB51_length_749_mean_cov_10.6608811749**

```
AAGACACCAACGCCCACACGAAATAGAAACCAGAAC|GAAAGAAAAAAAACAAAAAAAAAAAAAAAACAA|TTTGGGAGGCCGAGGCAG
                                     >chr16:60909502-60909535 - E=5e-02 p=0e+00
GTGGATCACAAGGTCAGGAGATCGAGACCATCCTGGCTAACATGGTGAAACCCCG|TCTCTACTAAAAATACAAAAAATTAGCCAGGCG
                                                        >chr9:121422411-121422703 - E=1e
TGGTGGCGGGCGCCTGTAGTCCCAGCTACTTGGGAGGCTGAGGCAGGAAAATCACTTGAACCCGGGAGGTGGAGCTTGCAGTGAGGCGA
-112
GATCACGCCACTGCACTCCAGCCTGGGCGACAGAGTGACTCCTTCTCAAAGATCAAATAAAATAATTAAAAATATATATATTATTCAGG

CCTAGTCCTTAGAAATAATACTTGTCCATAGACTAGCACATAGAAATAATATCATTAATATTTATAATATATGAAT|AAC|ATATAATG
                                                                             >chr9:1215
ATTTTGACACCTGAAATACCTATGCTGTCTCAGATGATGTCAGGGGAACAGTCTAATCCAAGCTTGTCCAACCCGTGGTCAGTGGGCCA
77063-121577385 - E=7e-176
CATTTGGCACAGGATGGCTGTGAATGCAGCCCAACACAAATTTGTAAACTTTCTTAAAACATTATGAGATTTTTTTACAGTTTTTTTTT

TTAGCTCATTAGTTATTGTTAGTATTAGTGTATTTTATGTATGGCTCAAGACAATTCTTCTTCCAATGTGGACCAGGGAAGCCAAAAGA

TTGGGCACTCCTGATCTAATGAGAATGGCAGTGTTCAGAAGT
```
